# Periplasmic expression of soluble single chain T cell receptors is rescued by the chaperone FkpA

**DOI:** 10.1186/1472-6750-10-8

**Published:** 2010-02-03

**Authors:** Kristin S Gunnarsen, Elin Lunde, Per E Kristiansen, Bjarne Bogen, Inger Sandlie, Geir Å Løset

**Affiliations:** 1 Centre for Immune Regulation, University of Oslo, Oslo, Norway; 2 Department of Molecular Biosciences, University of Oslo, N-0316 Oslo, Norway; 3 Institute of Immunology, Rikshospitalet University Hospital, N-0027 Oslo, Norway

## Abstract

**Background:**

Efficient expression systems exist for antibody (Ab) molecules, which allow for characterization of large numbers of individual Ab variants. In contrast, such expression systems have been lacking for soluble T cell receptors (TCRs). Attempts to generate bacterial systems have generally resulted in low yields and material which is prone to aggregation and proteolysis. Here we present an optimized periplasmic bacterial expression system for soluble single chain (sc) TCRs.

**Results:**

The effect of 1) over-expression of the periplasmic chaperon FkpA, 2) culture conditions and 3) molecular design was investigated. Elevated levels of FkpA allowed periplasmic soluble scTCR expression, presumably by preventing premature aggregation and inclusion body formation. Periplasmic expression enables disulphide bond formation, which is a prerequisite for the scTCR to reach its correct fold. It also enables quick and easy recovery of correctly folded protein without the need for time-consuming downstream processing. Expression without IPTG induction further improved the periplasmic expression yield, while addition of sucrose to the growth medium showed little effect. Shaker flask yield of mg levels of active purified material was obtained. The Vαβ domain orientation was far superior to the Vβα domain orientation regarding monomeric yield of functionally folded molecules.

**Conclusion:**

The general expression regime presented here allows for rapid production of soluble scTCRs and is applicable for 1) high yield recovery sufficient for biophysical characterization and 2) high throughput screening of such molecules following molecular engineering.

## Background

The antigen (Ag) specific receptor of the T cell lineage, the TCR, is a transmembrane heterodimer of covalently coupled α- and β-polypeptide chains. Each chain consists of two extracellular immunoglobulin (Ig) domains, one variable (V) and one constant (C), and the two V domains comprise the ligand binding portion that specifically interacts with a peptide/major histocompatibility complex (pMHC). TCRs are detection molecules with exquisite specificity and exhibit, like Abs, an enormous diversity. The fine tuning of the specificity, MHC restriction and thymic selection is incompletely understood. However, recent re-evaluation and comparison of the existing pMHC/TCR crystal structures demonstrate conservation of specific TCR-MHC contacts in complexes bearing common V segments and MHC allotypes. This has for the first time made it possible to postulate a TCR-MHC recognition code [1-5]. However, the predictions are still based on a restricted number of crystallographic data sets primarily due to the lack of a robust and versatile expression system for soluble TCRs necessary for obtaining sufficient amounts of protein. Soluble TCRs are prone to aggregation and missfolding, and many of the difficulties encountered may probably be explained by the fact that TCRs, unlike Abs, have not evolved to be secreted, but are expressed as membrane-bound molecules that are intrinsically unstable when expressed as soluble molecules.

Several engineering strategies for making soluble versions of TCRs, including scTCRs [6-8] and fusion of the extracellular TCR domains to other proteins; i.e. maltose binding protein, human constrant kappa domain (huCκ) or leucine zippers [9-16] have been reported. However, all of these strategies have shown limited success due to low production yield, poor functionality, or lack of crystallization abilities [17,18]. The introduction of a non-native disulphide bond in the TCR invariant region, to make so called dsTCR, has greatly increased the stability and folding characteristics of more than 20 human TCRs when expressed as cytosolic inclusion bodies that have been refolded [19]. Even without this artificial disulphide bond, optimized bacterial inclusion body expression and refolding has so far shown the highest success rate for obtaining soluble TCRs in high yields [20]. Recently an improved strategy for soluble periplasmic *E. coli *expression based on rational mutagenesis, over-expression of Skp, and fusion to the Ab C_κ _domain, was reported [9]. However, all of the expression systems described so far represents labour intensive and low through-put avenues that require either inclusion body denaturation and refolding [17], introduction of solubility-increasing amino acid substitutions or fusion to a second protein which might interfere with downstream applications [9].

The extracellular part of TCRs and Ab Fab fragments are structurally similar. This is also the case for molecules that consist of the two V domains connected by a flexible linker, namely single chain fragment variable (scFv) and scTCRs, respectively. *E. coli *expression of Ab derived fragments has been highly successful and a number of vector systems and expression strategies exist ([21] and references herein). These are to a large extent based on direct targeting to the periplasm with or without co-expression of chaperones, such as *skp*, *fkp, trigger factor *and *dsb*C [9,22-31].

In the current report, we describe an improved periplasmic expression system that allows rapid expression of unmodified soluble scTCRs based on over-expression of FkpA. The expression system was developed in *E. coli *XL1-Blue to complement a recently reported TCR phage display platform based on this particular host [32]. A systematic evaluation of a variety of parameters known to influence heterologous protein expression in *E. coli *was conducted using two scTCRs derived from the T cell clones 4B2A1 and 7A10B2 associated with a mouse model for idiotype (Id) dependent immune regulation [33-35]. Notably, these scTCRs are genetically unrelated. The final expression and purification protocol allowed the isolation of monomeric soluble scTCRs, with correct native fold and apparent functional activity. Key advantages over earlier TCR expression systems are the ability to produce "native" TCR sequences without previous engineering steps such as the introduction of solubility increasing mutations or fusion proteins and the ability to generate soluble material without refolding strategies. This system can therefore be used as a soluble expression platform downstream of a phage display library selection, or as an initial screen of a panel of native TCR clones to pick out the best starting point before stability or affinity maturation engineering.

## Methods

### Abs and additional reagents

The mouse anti-HIS-HRP Ab was purchased from AbD Serotec (Oxford, UK). The anti-FLAG M2-HRP Ab was purchased from Sigma-Aldrich (Oslo, Norway). All restriction enzymes were purchased from New England Biolabs (Ipswich, MA, USA). TCR-specific mAbs were GB113 [36] (clonotype-specific for 4B2A1), F23.1 [37] (recognizes TRBV13), 44-22-1 [38] (recognizes TRBV19), RR4-7 [39] (recognizes TRBV19). F23.1 and 44-22-1 were kind gifts from Dr. Uwe D. Staerz (Department of Medicine, National Jewish Medical and Research Center, Denver, USA) and Dr. Hans Hengartner (Institute for Experimental Immunology, University Hospital Zurich, Zurich, Switzerland), respectively. RR4-7 was purchased from BD Pharmingen (San Diego, CA, USA). The scFv control was a kind gift form Affitech AS (Oslo, Norway).

### T cell Clones

4B2A1 and 7A10B2 T cell clones are specific for residues 91-101 of mouse Ig λ2^315 ^light chain presented on the MHC class II I-E^d ^molecule [40,41]. The clones are distinguished by the 4B2A1 not cross-reacting to a Phe^94 ^(mutated λ2^315 ^sequence) to Tyr^94 ^(germline λ2) substitution while 7A10B2 does [42]. Moreover, while 4B2A1 expresses a unique TCR [TRAV7D-3*01, TRAJ40*01/TRBV13-2*01, TRBD1*01, TRBJ1-2*01], 7A10B2 expresses a dominant and recurrent TCR [TRAV9-3*01, TRAJ58*01/TRBV19*01, TRBD1*01, TRBJ1-1*01] in this system [43].

### Plasmids and bacterial strains

The soluble expression vectors pHOG-Dummy [44] and pHOG21 [45] were a kind gift from Affitech AS (Oslo, Norway). pHOG21 encodes a scFv with specificity against 2-phenyloxazol-5-one (phOx) coupled to bovine serum albumin (BSA), originally isolated from a human Ab phage library [46]. The pFKPDN phagemid encodes either the 4B2A1 [TRAV7D-3*01, TRAJ40*01/TRBV13-2*01, TRBD1*01, TRBJ1-2*01] or 7A10B2 [TRAV9-3*01, TRAJ58*01/TRBV19*01, TRBD1*01, TRBJ1-1*01] scTCRs that have been described in detail previously [32]. *E. coli *XL-1 Blue (*recA1 endA1 gryA96 thi-1 hsdR17 supE44 relA1 lac [F' proAB lacI^q^ZΔM15 Tn10 (Tet^r^)] *was purchased from Stratagene (LaJolla, CA, USA).

### Construction of the scTCR pHOG, pFKPEN and pFKPEI expression vectors

**Figure 1:**
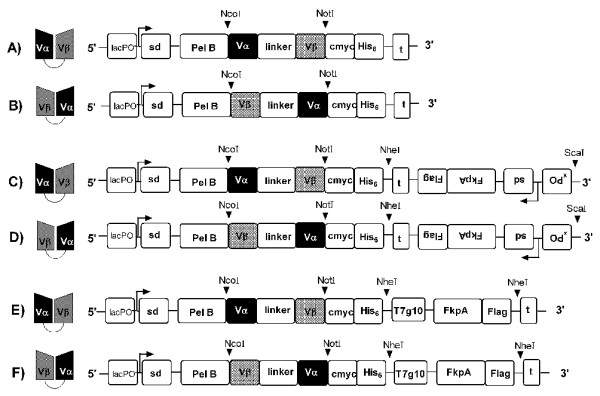
**Schematic drawing of the scTCR molecules (left) and scTCR encoding vector constructs (right)**. (A) and (B) scTCR Vαβ and Vβα in pHOG. (C) and (D) scTCR Vαβ and scTCR Vβα in pFKPEN. (E) and (F) scTCR Vαβ and Vβα in pFKPEI. Abbreviations: lacPO, lac promoter, sd, Shine-Dalgarno sequence, T7g10, translation initiation region, t, T7 transcription terminator, xPO, native *fkpA *promotor, NcoI, NotI, NheI and ScaI, Restriction enzyme-sites.

The pHOG expression vectors (Figure 1A and 1B) were generated by subcloning the scTCR encoding cassettes from pFKPDN phagmid into pHOG on compatible *Nco*I/*Not*I RE sites using standard techniques, before transformation into *E. coli *XL1-Blue cells. Likewise, the pFKPEN expression vectors (Figure 1C and 1D) were created by subcloning the FkpA expression cassette from pFKPDN into these new vectors on compatible *Sca*I/*Nhe*I RE sites. The *fkpA *gene is constitutively expressed from its native promoter, xPO, in pFKPEN (Figure 1C and 1D).

To generate an expression vector were FkpA transcription is controlled by the lac promoter that also controls the scTCR transcription, the pFKPEI expression vector was made (Figure 1E and 1F). The genomic *E. coli *K12 sequence (Accession no.: NC_000913) was used for primer design to isolate the *fkpA *gene ORF. The forward primer was designed with a 5'-end *Nhe*I tag followed by the T7g10 TIR region [47] and the *fkpA*-encoding portion (Additional file 1). The reverse primer also included a 5'-end *Nhel*I RE site and a FLAG tag encoding sequence (Additional file 1). PCR was applied to 1 μl of *E. coli *XL1-Blue overnight culture using Vent DNA polymerase (New England Biolabs, Ipswich, MA, USA). The resulting DNA fragment was ligated into pHOG-Dummy [32] on the corresponding *Nhe*I RE site creating the new pFKPEI-Dummy expression vector. To generate the final pFKPEI expression vector (Figure 1E and 1F), the *Nco*I/*Not*I scTCR segments were cloned into this construct as described above, before transformation into *E. coli *XL1-Blue cells. The pFKPEI expression vector (Figure 1E and 1F) contains a dicistronic expression cassette with the scTCR open reading frame (ORF) followed by a translation initiation region (TIR) and the FkpA ORF, both controlled by the lac promoter. All the constructs were validated by RE analysis and subsequently confirmed by sequencing.

### Protein production

*E. coli *XL1-Blue cells transformed with the plasmids were inoculated from glycerol stocks into 5 ml LB-medium supplemented with 30 μg/ml tetracycline, 100 μg/ml ampicillin and 0.1 M glucose (LB_TAG _medium), in 50 ml tubes and incubated at 37°C/220-rpm overnight (ON). The cultures were then re-inoculated into 5 ml LB_TAG _medium at an OD_600 nm _of 0.025 in 50 ml tubes and incubated at 37°C/220 rpm. At OD_600 nm _0.6-0.8, the bacteria were pelleted (3600-g/10 min/RT) and the pellets resuspended in 5 ml LB medium supplemented with 0.1 mM isopropyl-β-D-thio-galactopyranoside (IPTG), 30 μg/ml tetracycline and 100 μg/ml ampicillin. Cultures were then incubated at 30°C/250 rpm/ON. To directly compare specific scTCR and FkpA protein production from each clone, cell numbers were normalized according to OD_600 nm _before being channelled into an identical cellular compartment fractionation procedure.

The standard expression conditions described above were varied such that the cultures were grown under standard conditions until OD_600 nm _0.6-0.8, pelleted and resuspended in LB-medium supplemented with the following additives, either 0.1 mM IPTG and 0.4 M sucrose, 0.4 M sucrose or without any additives. Again, cell numbers were normalized as above. An up-scaled shaker flask production was performed in 400 ml expression cultures without any additives for 4B2A1 scTCR Vαβ in pFKPEI, while 4B2A1 scTCR Vβα in pFKPEI was performed with 0.1 mM IPTG additive, in 2 L shaker flasks. 7A10B2 scTCR Vαβ and Vβα in pFKPEN was produced in several small scale cultures (10 × 50 ml) without any additives. All cultures were then subject to the cellular compartment fractionation procedure.

### Cellular compartment fractionation

Cells were harvested by centrifugation at 15 min/4600-g/4°C and separated into; (1) medium fraction, (2) soluble periplasmic fraction and (3) cytosolic fraction by a modified version of that described by Kipriyanov et al. [45]. Briefly, the medium fraction was obtained by an additional centrifugation of 1 ml supernatant (10 min/16,530-g/4°C). To obtain the cytosolic and periplasmic fractions, the pellet was resuspended in 490 μl ice-cold periplasmic extraction solution (50 mM Tris/HCl, 20% sucrose, 1 mM EDTA, pH 8) supplemented with 5 μl lysozyme (100 mg/ml in 50 mM Tris/HCl, pH 8) and 5 μl RNase A (10 mg/ml in50 mM Tris/HCl, pH8) before incubation for 1 h at 4°C with rotation. The mixture was centrifuged, leaving the soluble periplasmic fraction as the supernatant. The pellet, containing the cytosolic fraction, was resuspended in 475 μl ice-cold 0.1 M Tris-HCl, pH 8, before addition of 167 μl 4× sample buffer (200 mM Tris-HCl, 8% SDS and 40% glycerol) and subjected to rough pipetting to dissolve the pellet. For the large scale (400-500 ml) cultures, the same fractionation procedure was used after adjusting the reagent volumes × 80.

### Protein purification

The periplasmic fractions isolated after 400-ml expression were diluted 1:3 in binding buffer (40 mM sodium phosphate, 0.5 M NaCl, 40 mM imidazole, pH 7.4) and filtered through a 0.2 μm sterile filter (Millipore). The samples were purified by immobilized metal affinity chromatography (IMAC) on a His-TrapHP column (GE Healthcare Life Sciences) as described in the protocol provided by the manufacturer. The eluted protein fractions were pooled, concentrated by Amicon Ultra-15 Centrifugal Filter Units (Millipore) and dialyzed against storage buffer (1× PBS, 500 mM NaCl, pH 6.5). Protein concentration was determined by A_280 nm _on a NanoDrop ND-1000 apparatus (Thermo Fischer Scientific Inc., Walmington, USA) based on calculated extinction coefficients [48]. Size exclusion chromatography (Superdex 75 10/300 GL, GE Healthcare Life Sciences) was performed on an automated ΔKTA chromatography system, (GE Healthcare Life Sciences) using 70 μl of the IMAC purified protein to isolate monomeric scTCR. The concentration of protein in each fraction was determined as described above, followed by analysis on non-reducing and reducing SDS-PAGE and western blotting as described below. The monomeric fractions were pooled, concentrated by Amicon Ultra-4 Centrifugal Filter Units (Millipore) followed by concentration determination as before.

### SDS-PAGE and western blotting

Identical volumes of fractionated samples isolated in parallel from A_600 nm _normalized *E. coli *cell cultures were separated by non-reducing SDS-PAGE on 4-12% Bis-Tris XT precast gels (Bio-Rad, Hercules, CA, USA) and blotted onto a polyvinylidene fluoride membrane (Millipore, Madison, USA) in Tris/glycine buffer (25 mM Tris, 192 mM Glycine, and 20% methanol, ph 8.3) at 25 V for 30 min using a semi-dry blotting apparatus (Bio-Rad, Hercules, CA, USA). The membrane was blocked in PBSTM (1× PBS supplemented with 0.05% v/v Tween 20 and 4% w/v skim milk) before scTCR proteins were detected with anti His-HRP Ab (1:10, 000). The membrane was washed and developed with SuperSignal™ West Pico substrate (Pierce, Rockford, IL, USA) before exposed to BioMax MR film (Kodak, Fernwald, Germany). Expression of rFkpA was detected after 30 min stripping by Restore™ Western Blot Stripping Buffer (Pierce, Rockford, IL, USA), and the membrane redeveloped as above using an anti-FLAG M2-HRP Ab (1:10 000). Samples of 0.25 μg Superdex 75 purified scTCRs were analyzed in parallel with 0.25 μg IMAC purified scTCRs by non-reducing and reducing SDS-PAGE followed by western blotting as described above.

### scTCR capture ELISA

The various mAbs were absorbed to MaxiSorp microtiter plate wells (Nunc, Roskilde, Denmark) at a concentration of 5 μg/ml in 1 × PBS, pH 7.4 ON at 4°C. The wells were blocked with PBSTM for 1 h at RT, and IMAC purified scTCR samples at a concentration of 4 μg/well and Superdex75 purified scTCR samples at a concentration of 0.4 μg/well were added. All were allowed to react for 2 h at RT before the captured scTCRs were detected with anti-His-HRP Ab (1:5000) for 2 h at RT. The wells were developed with 100 μl of TMB soluble substrate (Calbiochem), stopped by adding 1 M HCl after 30 min, and the absorbance read at A_450 nm_.

### Circular dichroism (CD) spectroscopy

CD spectra were recorded using a Jasco J-810 spectropolarimeter (Jasco International Co., Ltd., Tokyo Japan). Measurements were performed at 25°C using a quartz cuvette (Starna, Essex, UK) with a path length of 0.1 cm. Measurements were performed with a protein concentration of 0.120 mg/ml for 4B2A1 scTCR Vαβ and 0.150 mg/ml for the control scFv Ab fragment in 1× PBS, supplemented with 500 mM NaCl (pH 6.4). Samples were scanned 5 times at 50 nm/min, using a band width of 1 nm. The response time was 2 s, and the wave length range was 200-260 nm. The data were averaged and the spectrum of a sample-free control was subtracted. The secondary structure elements of the proteins were calculated from ellipticity data, using the spectral fitting methods, CONTIN/LL [49], SELCON3 [50] and CDSSTR [51], in the CDpro package [52]. Thermal denaturation was determined as the change in CD signal from heating. The temperature was controlled with a TPC-423S/L system (Jasco International Co).

### Secondary structure prediction

Secondary structure element of the 2C TCR structure (PDB:2oi9) [53] were predicted using the DSSP [54] and Stride[55].

## Results

### Construct design

The pHOG (Figure 1A, B), pFKPEN (Figure 1C, D) and pFKPEI (Figure 1E, F) vectors drive scTCR expression from the lac promoter. In all cases, the scTCRs are fused to an *N *-terminal pelB signal sequence that directs protein translocation across the inner membrane of *E. coli *through the SecB-dependent pathway to the oxidising environment of the periplasm. This oxidising environment is a prerequisite for generation of disulphide bridges within proteins such as scTCRs [56,57]. The pFKPEN and pFKPEI vectors also carry the gene encoding the periplasmic chaperone FkpA. In pFKPEN, recombinant FkpA (rFkpA) expression is controlled by its native promoter, while pFKPEI contains a dicistronic expression cassette where rFkpA gene transcription is controlled by the lac promoter.

In all vectors, the scTCR cassette was constructed by genetically fusing V_α _and V_β _encoding gene segments with a sequence encoding a synthetic linker [44]. To gain insight into how domain orientation affects soluble expression, two versions with alternative domain orientation were made, such that either V_α _(Figure 1A, C and 1E) or V_β _(Figure 1B, D and 1F) was expressed *N*-terminally. All scTCRs were fused to a c-Myc- and a His-tag *C*-terminally, whilst rFkpA was fused to a *C*-terminal FLAG-tag. All constructs used in the current study are described in Figure 1.

### Expression level and subcellular localization

**Figure 2:**
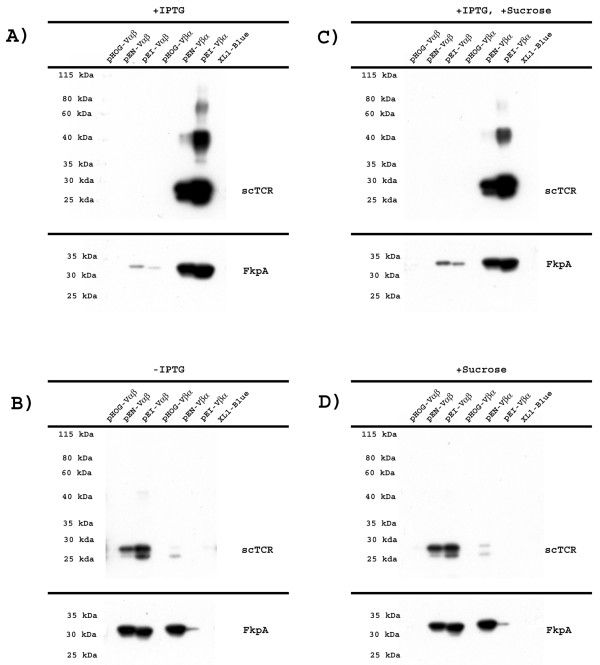
**4B2A1 scTCR and rFkpA expression level**. Western blot showing periplasmic expression levels of 4B2A1 scTCR variants and rFkpA from pHOG, pFKPEN (pEN) and pFKPEI (pEI) vectors under different expression conditions, detected with anti-His tag -HRP Ab and anti-Flag- M2 HRP Ab as described. The scTCR and rFkpA were developed with SuperSignal West Pico substrate and exposed to BioMax MR film for for 30 min and 30 sec, respectively, for the upper and lower panels. The expression analysis was repeated twice, and representative results are shown.

**Figure 3:**
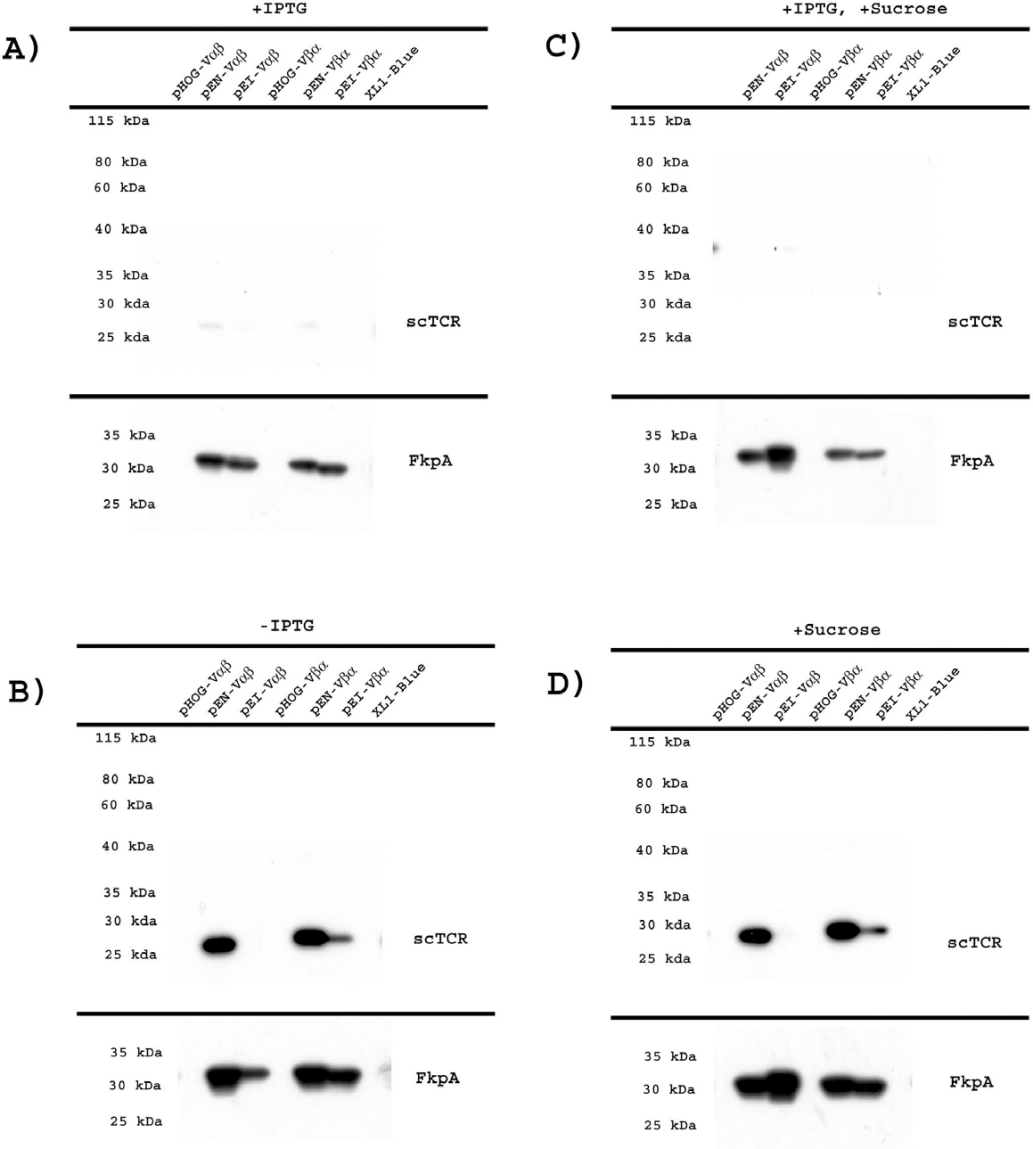
**7A10B2 scTCR and rFkpA expression level**. Western blot showing periplasmic expression levels of 7A10B2 scTCR variants and rFkpA from pHOG, pFKPEN (pEN) and pFKPEI (pEI) vectors under different expression conditions, detected with anti-His tag-HRP and anti-Flag-M2-HRP as described. The scTCR and rFkpA were developed with SuperSignal West Pico substrate and exposed to BioMax MR film for for 30 min and 30 sec, respectively, for the upper and lower panels. The expression analysis was repeated twice, and representative results are shown.

Expression was performed in *E. coli *XL1-Blue followed by cell fractionation and periplasmic expression analyzed by SDS-PAGE and Western blotting, as described in the Method section. As shown in Figure 2, 4B2A1 scTCR was not detected in the periplasmic fraction using anti-tag mAbs in the absence of rFkpA over-expression (pHOG-plasmids), regardless of domain orientation and expression conditions. The 7A10B2 scTCR behaved like 4A10B2 (Figure 3). Thus, both scTCRs failed to reach the periplasmic space, and rather aggregated in the cytosol (results not shown). The growth characteristics of each culture were studied and the results are summarized in Table 1. The one expressing 4B2A1 Vαβ scTCR showed sign of growth arrest. All others demonstrated overall robust growth and reached a final cell density similar to that of the XL1-Blue control without plasmid.

**Table 1:** Growth characteristics^a^

TCR format	Vector	+IPTG	-IPTG	+Sucrose	+IPTG +Sucrose
4B2A1 scTCR Vαβ	pHOG	0.6^b^	2.6	1.36	0.48^b^
	pFKPEN	3	2.8	2.2	2.2
	pFKPEI	2.8	2.8	2.1	2.4

4B2A1 scTCR Vβα	pHOG	2.6	2.6	2.2	2.2
	pFKPEN	2.8	3	2.5	2.4
	pFKPEI	2.7	2.8	2.2	2.2

7A10B2 scTCR Vαβ	pHOG	3	3	2.6	2.4
	pFKPEN	0.8^b^	2.8	2.2	0.58^b^
	pFKPEI	2.8	2.6	1.8	2

7A10B2 scTCR Vβα	pHOG	3	3	2.4	0.48^b^
	pFKPEN	2.8	2.8	2.3	2.6
	pFKPEI	2.8	2.8	2	2.2

XL1-Blue cells without plasmid		2.8	2.8	2	2.2

^a ^A_ 600 nm _after ON incubation of 5 ml expression cultures

^b ^No growth of cultures after induction

The chaperone FkpA has a highly beneficial effect on periplasmic expression and correct folding of toxic Ab scFvs, [27], and we have recently reported a similar effect on phage display of scTCRs [32]. Thus, the effect of FkpA over-expression was assessed here. The clones that were picked for further study, all expressed high levels of FkpA as demonstrated by SDS-PAGE and western blot of whole cell lysates using anti-Flag Ab (results not shown).

The periplasmic levels of scTCRs in both configurations, Vαβ and Vβα, were detected under different expression conditions. The levels of rFkpA in the periplasm were also assessed. When expression was induced with IPTG, a high yield of 4B2A1 scTCR in Vβα orientation was observed, while scTCR with the opposite Vαβ orientation was not (Figure 2A). The highest yield of 4B2A1 Vβα scTCR was obtained when rFkpA transcription was under lac promoter control. All four of these 4B2A1 scTCR producing cultures (Vαβ and Vβα from pFKPEN and pFKPEI) showed robust growth (Table 1). The 7A10B2 scTCR was not detected or detected in negligible amounts in the periplasmic fraction regardless of orientation (Figure 3A). The scTCR Vαβ producing culture showed sign of growth arrest when rFkpA was transcribed from its endogenous promoter (Table 1).

It has earlier been reported that a reduction of the IPTG concentration has a positive effect on periplasmic yield of soluble protein expressed from a lac promoter [45]. Indeed, when expression was performed without IPTG, the periplasmic yield of 4B2A1 Vαβ increased considerably. The yield of 4B2A21 Vβα, on the other hand, decreased (Figure 2B). The yield of 7A10B2 scTCR in both domain orientations increased (Figure 3B). All cultures reached high and similar final cell density (Table 1). Analysing periplasmic FkpA levels, this was equal to or higher than that observed in the presence of IPTG in all cases, except when expressed from the lac promoter in the vector encoding 4B2A1 scTCR Vβα. This was in line with the fact that very little 4B2A1 scTCR Vβα was found in the periplasmic space in the absence of induction.

Addition of 0.4 M sucrose to the growth medium has been shown to increase the yield of secreted proteins, either in the periplasm or culture medium [45,58]. To investigate if the soluble scTCR yield in the periplasmic fraction could be further increased, the effect of sucrose addition was assessed both in uninduced and IPTG induced cultures. Neither the 4B2A1 (Figure 2C and 2D) nor 7A10B2 (Figure 3C and 3D) scTCR levels increased after sucrose addition. The expression patterns were identical to that observed without sucrose addition, and no increased leakage to the culture medium was observed (data not shown).

Taken together, overall good yields of periplasmic 4B2A1 scTCR were seen in the presence of periplasmic rFkpA expression. This was observed from both pFKPEN and pFKPEI plasmids; for Vαβ, when expression was performed in the absence of IPTG and for Vβα, when expression was performed in the presence of IPTG. For 7A10B2 scTCR, the overall highest yield was seen from the pFKPEN plasmid in the absence of IPTG for both the Vαβ and the Vβα configuration.

### Protein purification

**Figure 4:**
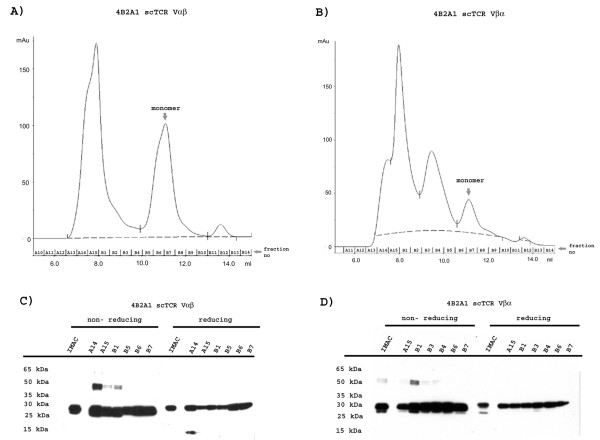
**Purity of proteins**. (A) and (B); S75 size exclusion chromatography of scTCR 4B2A1 Vαβ and Vβα respectively. 50 μl of the IMAC purified material was subject to the purification procedure. In (A) fractions A14-B1 corresponds to the higher aggregates, while fraction B5-B7 corresponds to the monomeric scTCR 4B2A1 Vαβ, the monomeric fraction is highlighted with an arrow. In (B) fraction A15-B1 and B3-B4 corresponds to the higher aggregates, while fraction B6-B7 corresponds to the monomeric scTCR 4B2A1 Vβα, the monomeric fraction is highlighted with an arrow. (C) and (D); Western blots showing reduced and non-reduced scTCR Vαβ and Vβα after IMAC purification and S75 size exclusion chromatography. Samples of 0.25 μg were loaded on the gel and analysed with anti-His tag-HRP.

The four scTCR variants, 4B2A1 and 7A10B2 in both domain orientations, were expressed at large scale under conditions shown to give good small scale periplasmic yields. Protein fractionation was performed as described above, followed by IMAC purification. Eluted fractions containing protein were pooled and concentrated. The production yields after concentration is given in Table 2 and were 3.6 and 3.9 mg/l for the 4B2A1 Vαβ and Vβα variants, respectively. Very low yield of the 7A10B2 variants were obtained. Thus, the two 4B2A1 samples only were further analyzed by size exclusion chromatography (Superdex 75 GL 10/300) (Figure 4A and 4B), followed by non-reducing and reducing SDS-PAGE of eluted fractions (Figure 4C and 4D). Importantly, the Vαβ format had more than double the amount of monomeric scTCR compared to the Vβα format (Figure 4A and 4B, and table 2). However, both formats were produced as polymers that appeared to be composed of both non-covalent and covalent aggregates (Figure 4C and 4D). Taken together, the overall best yield of monomeric scTCR was from the 4B2A1 clone in the Vαβ orientation. This format yielded 2.1 mg per L culture of active purified monomeric material, which is comparable with or better than amounts acquired by others using periplasmic expression systems (table 2) [26,9]. Importantly, the huCκ fusion format reported by Maynard et al [9] requires downstream proteolytically removal of the huCκ moiety, hence the actual soluble scTCR yield will be equal to or lower than with our approach. Moreover, the absence of this huCκ fusion resulted in wastely reduced scTCR yield (table 2) [9].

**Table 2:** Production yield

TCR format	Total soluble production yield mg/L culture^a^	Soluble monomeric production yield mg/L culture^b^
4B2A1 scTCR Vαβ	3.6	2.1
4B2A1 scTCR Vβα	3.9	0.44
7A10B2 scTCR Vαβ	0.78	ND
7A10B2 scTCR Vβα	0.24	ND

1934^c^	ND	3.8
c19^c^	ND	3
2B4^c^	ND	0.5
2B4^d^	ND	0.015

^a ^Estimated periplasmic yield of total soluble scTCRs after IMAC purification.

^b ^Estimated periplasmic yield of monomeric soluble scTCR after size exclusion chromatography.

^c ^Values implemented from Maynard *et al *[9], scTCR-human C kappa fusion format

^d ^Values implemented from Maynard *et al *[9], scTCR format

ND; Not determined

### Folding and secondary structure analysis

#### ELISA

The conformational integrity of the IMAC and size exclusion chromatography purified 4B2A1 scTCRs was analyzed using two V-gene specific mAbs in a capture ELISA. Furthermore, 7A10B2 scTCRs were produced in several parallel small scale cultures, that were pooled, IMAC purified and analyzed in capture ELISA.

**Figure 5:**
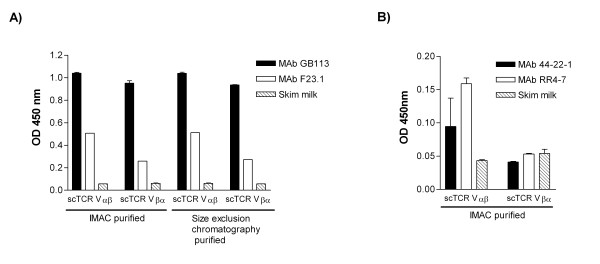
**Integrity analysis of scTCR**. A) ELISA analysis of equal amount of purified 4B2A1 scTCR captured on TCR V-gene specific mAbs. B) ELISA analysis of equal amount of purified 7A10B2 scTCR captured on TCR V-gene specific MAbs. One of two independent experiments performed in duplicates is shown with the SD indicated as error bars.

The results show that differences exist in mAb reactivity between the scTCRs with regard to domain orientation. For both 4B2A1 and 7A10B2 scTCR, the Vαβ scTCRs exhibited stronger mAb reacitivity than the Vβα counterparts (Figure 5A and 5B). Both anti-Vβ8 MAb F23.1 and clonotype-specific GB113 bound the purified 4B2A1 scTCRs fractions (Figure 5A). GB113 bound the two molecules equally well, whilst F23.1 bound better to Vαβ than to Vβα, Likewise, the IMAC purified 7A10B2 Vαβ scTCR was detected by the Vβ6-specific 44-2-1 and RR4-7 MAbs, whereas no binding was observed in the Vβα domain orientation (Figure 5B).

#### Structural analysis by CD spectroscopy

**Figure 6:**
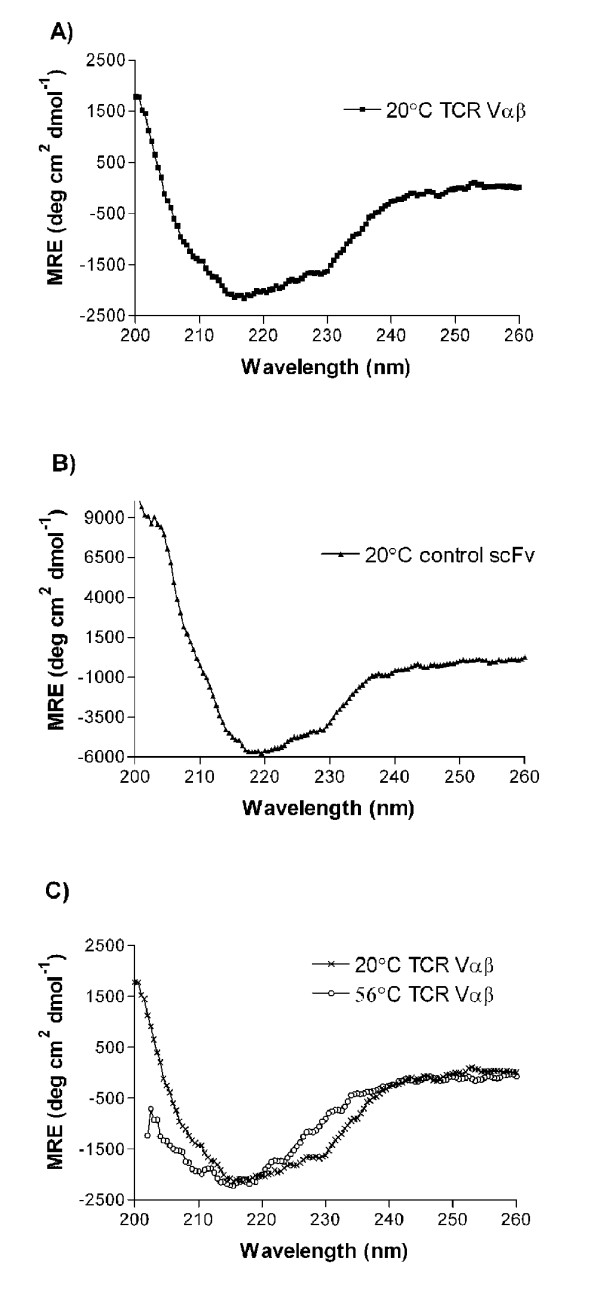
**Circular dichroism spectra of the purified scTCR Vαβ protein (A) and a control scFv Ab fragment (B) collected at 20°C**. CD scans at two different temperatures, 20°C and 56°, are shown in (C). MRE, mean residual ellipicity.

scTCR Vαβ 4B2A1 and a control scFv Ab fragment were subjected to CD spectroscopy at 20°C and 56°C as shown in Figure 6, and the spectra obtained analyzed using CONTIN/LL [49], SELCON3 [50] and CDSSTR [51]. The obtained structure data from the three fitting techniques were similar. However, CONTIN/LL gave slightly lower RMSD ratios than CDSSTR. The results from CONTIN/LL are thus given in Table 3. The CD data analysis indicated a 47% and 42% β-sheet content as well as a 5% and 5% α-helix content in the scFv control and 4B2A1 scTCR Vαβ molecules, respectively. The spectra of both 4B2A1 scTCR Vαβ (Figure 6A) and the control scFv Ab fragment (Figure 6B) are hence typical of a protein with high degree of β-sheet structure characteristic for the Ig-fold. This is consistent with the secondary structure element prediction given by DSSP [54] and Stride [55] for the 2C TCR structure (PDB: 2oi9) [53], which calculates a 49% β-sheet content and a α-helix content of 4.5%. The degree of secondary structure content decreased as the temperature was raised towards 56°C as seen in Table 3 and Figure 6C, indicating thermally induced unfolding of the protein.

**Table 3:** Secondary structure elements^a^

	scFv at 20°C	scTCR at 20°C	scTCR at 56°C
α-Helix	5	5	5
β-Sheet	47	42	20
β-Turn	20	21	20
Random coil	28	32	39

^a ^The secondary structure elements were estimated by CONTIN/LL from the CD spectrums presented in Figure 6. The experiment was repeated twice, and representative results are shown.

## Discussion

The aim of the current study was to generate a general expression system for soluble scTCRs. The 4B2A1 and 7A10B2 scTCRs used have previously been successfully expressed as fusions to pIII on filamentous phage [32]. Based on that study, the current report focuses on the soluble scTCR format and investigation of protein yield and sub-cellular localization using a bacterial system compatible with the *E. coli *host used in scTCR phage display, namely XL1-Blue.

Chaperones are known to enhance expression yields as they facilitate folding, prevent aggregation, reactivate aggregates and reduce protein degradation [28,59-61]. Bothmann and Plückthun [23,27] identified two periplasmic factors, namely FkpA and Skp, which increased the functional periplasmic yields of toxic Ab scFvs, while already well-expressing clones were unaffected. FkpA is a periplasmic peptidyl-propyl *cis,trans*-isomerase, but the effect was also striking for scFvs that did not contain *cis*-prolines. The positive effect rather seemed to be a result of the ability of the chaperone to prevent premature aggregation of early folding intermediates as well as reactivation of inactive proteins [28,62]. Skp belongs to a family of cavity-containing chaperons and works by protecting its substrate from aggregation within this cavity [63]. Skp was recently reported to have an effect on scTCR expression [9]. However, co-expression of Skp and FkpA showed no synergistic effect on scFv yield. Rather, co-expression had the same effect as FkpA alone [27]. We have previously reported that over-expression of rFkpA normalized the display level of scTCRs-pIII fusions in phage display, while Skp had only minor effects [32]. In the present study, we therefore focused on FkpA co-expression.

Without chaperone co-expression, the scTCRs were trapped in the cytosol despite the fact that all constructs were equipped with a pelB leader sequence directing transport to the periplasm. When FkpA was co-expressed, we observed a positive effect on growth rate. Moreover, a profound effect on the accumulation of soluble 4B2A1 and 7A10B2 scTCRs in the periplasm was also seen. This was the case whether rFkpA was expressed from its native promoter or the lac promoter. These initial results hence showed that FkpA has an ability to significantly increase the periplasmic expression yield of the TCRs.

The effect of domain orientation on functional scTCR fragments has been reported earlier, and show the same trend as we see here, namely that the Vα-linker-Vβ domain orientation increases the functional fraction of scTCR fragments [32,64]. Generally, the most unstable fragment should be put *N*-terminally and the most stable fragment *C*-terminally. This prevents rapid folding of the *N*-terminal domain followed by abortive periplasmic translocation caused by a partly unfolded *C*-terminal domain [32,64]. The TRBV13-2 segment used in the 4B2A1 scTCR, is considered a particularly stable fragment [65,66]. The results in this report hence fit well with the results obtained by others.

Culture conditions, such as induction and growth in the presence of certain sugars, have been shown to influence the production yield and sub-cellular localization of proteins [45,58,67]. We therefore investigated the effect of sucrose addition to the medium as well as the effect of adding or removing IPTG. The results showed that 4B2A1 TCR in the Vβα orientation was expressed well under standard conditions, that is, in the presence of IPTG, but without addition of sugars. In contrast, 4B2A1 TCR Vαβ and 7A10B2 in both domain orientations were expressed poorly. Removal of IPTG from the growth medium restored the expression of these three molecules. Without IPTG residual transcription still occurs through the leaky lac promoter, but with decreased translation and hence folding load. This was found to be beneficial for the scTCRs.

It has previously been reported that growing cells in the presence of certain sugars, raffinose or sucrose, can reduce the aggregation of recombinant proteins in the *E. coli*periplasm [45,58]. These sugars are small enough to diffuse through the inner membrane to the periplasm, causing an increase in the osmotic pressure resulting in enlargement of the periplasmic space, and hence a decrease in the local protein concentration [45,68]. However, the addition of sucrose had no effect on the yield of scTCR in the periplasm. Possibly, the over-expression of rFkpA prevents premature aggregation so efficiently that the addition of sucrose has little or no detectable effect.

When large scale expression using optimalized expression conditions for each of the scTCRs was performed mg/L amounts were obtained from both 4B2A1 scTCRs after purification. The 7A10B2 derived scTCRs showed an altered expression profil after up-scaling, which has also been reported by others for antibody scFv fragments [45]. However, by pooling the periplasmic fraction from several small scale cultures, yields of μg/L were obtained. Thus, the 4B2A1 derived molecules only were chosen for studies of how domain orientation influences the yield of the monomeric fraction as well as folding. We found that the scTCR with the Vαβ domain orientation had a larger monomeric peek than the one with the Vβα domain orientation. Aggregates appeared to be formed after misfolding due to erroneous disulphide bond formation as well as through surface exposed hydrophobic patches.

The affinity of the monomers for their cognate pMHC (λ2^315^/I-E^d^) ligand, is too low for detection by flow cytometry. We therefore used binding to several well characterized MAbs as an indirect measurement of structural integrity and functionality. The same MAbs have been used earlier to characterize the same scTCRs displayed on phage [32] or expressed as TCR-Ig fusions (manuscript submitted). In both cases, binding to MAbs correlated with the ability of the TCR moiety to bind I-E^d ^expressing murine A20 lymphoma cells that had been loaded with the cognate λ2^315 ^peptide, and binding required a multivalent format (three-five copies on phage and two copies in the TCR-Ig fusion) [32]. Importantly, the current report shows differences in MAb reactivity with regard to domain orientation, and the Vαβ format was superior to the Vβα format. Furthermore, the secondary structure data given by CD measurements shows that the scTCR Vαβ has a native-like fold that in conjunction with the binding activity of the MAbs strongly indicates functionality.

## Conclusion

The work demonstrated that the presence of the chaperone FkpA, expression conditions as well as domain orientation greatly influence the yield and sub-cellular localization of soluble TCRs. Over-expression of FkpA in the periplasm is a prerequisite for soluble periplasmic localization of the scTCR, and absence of inducer (IPTG) further improved the periplasmic yield of formats which were poorly expressed under standard conditions. Addition of sucrose to the medium did not influence the yield. Comparing scTCR molecular design, the format with Vαβ domain orientation gives a better expression profile with regard to monomeric protein yield than the Vβα format, and the binding to specific MAbs is stronger. Furthermore, the purified 4B2A1 scTCR Vαβ exhibited well resolved secondary structure elements typical for the Ig fold topology indicating a correctly folded TCR moiety. The final protein yields obtained using our optimized approach perform equal or better than previously reported periplasmic expression systems and this was achieved without fusions or aa modifications of the TCR moiety. We anticipate that this system may enable rapid production of a wide range of soluble TCRs, and thereby simplify the screening of a new generation of recombinant TCRs generated by protein engineering and display techniques such as phage-display.

## Authors' contributions

KSG did most of the experimental work, participated in the design of the study and drafted the manuscript. EL gave initial technical guidance. PEK gave guidance on the CD analysis. BB provided key reagents, participated in the design of the study and in drafting the manuscript. GÅL designed and constructed the expression vectors, supervised the study and participated in drafting and finalizing the manuscript. IS supervised the study, provided funding and finalized the manuscript. All authors approved the final manuscript.

## Supplementary Material

Additional file 1 **Primer overview**. Primers used to isolate the *fkpA *gene ORF. Click here for file
