# Supplementary material for: Periplasmic expression of soluble single chain T cell receptors is rescued by the chaperone FkpA
Source: BMC Biotechnol. 2010 Feb 3;10:8. doi: 10.1186/1472-6750-10-8 (PMC2834602; doi:10.1186/1472-6750-10-8)
Supplement: Additional file 1 — Primer overview. Primers used to isolate the fkpA gene ORF. [file 1472-6750-10-8-S1.DOC]

| **Additional file 1. Primer overview** | |
| --- | --- |
| Forward primer | 5’-AGAGAGGCTAGCAATAATTTTGTTTAACTTTAAGAAGGAGATATACATATGAAATCACTGTTTAAAGTAACGCTGCTG-3’ |
| Reverse primer | 5’-AGAGAGGCTAGCTCACTTGTCGTCATCGTCCTTGTAGTCTTTTTTAGCAGAATCTGCGGCTTTCG-3’ |
